# Tudor-SN Regulates Milk Synthesis and Proliferation of Bovine Mammary Epithelial Cells

**DOI:** 10.3390/ijms161226212

**Published:** 2015-12-16

**Authors:** Jinxia Ao, Chengjie Wei, Yu Si, Chaochao Luo, Wei Lv, Ye Lin, Yingjun Cui, Xuejun Gao

**Affiliations:** The Key Laboratory of Dairy Science of Education Ministry, Northeast Agricultural University, Harbin 150030, China; aojinxia@163.com (J.A.); chengjiewei@163.com (C.W.); siyushenghua@sina.com (Y.S.); luochaochao839505@163.com (C.L.); lvwei199063@163.com (W.L.); linlu516@163.com (Y.L.); c_yingjun@sina.com (Y.C.)

**Keywords:** Tudor-SN, milk synthesis, cell proliferation, Stat5, NFκB1

## Abstract

Tudor staphylococcal nuclease (Tudor-SN) is a highly conserved and ubiquitously expressed multifunctional protein, related to multiple and diverse cell type- and species-specific cellular processes. Studies have shown that Tudor-SN is mainly expressed in secretory cells, however knowledge of its role is limited. In our previous work, we found that the protein level of Tudor-SN was upregulated in the nucleus of bovine mammary epithelial cells (BMEC). In this study, we assessed the role of Tudor-SN in milk synthesis and cell proliferation of BMEC. We exploited gene overexpression and silencing methods, and found that Tudor-SN positively regulates milk synthesis and proliferation via Stat5a activation. Both amino acids (methionine) and estrogen triggered NFκB1 to bind to the gene promoters of Tudor-SN and Stat5a, and this enhanced the protein level and nuclear localization of Tudor-SN and p-Stat5a. Taken together, these results suggest the key role of Tudor-SN in the transcriptional regulation of milk synthesis and proliferation of BMEC under the stimulation of amino acids and hormones.

## 1. Introduction

Tudor staphylococcal nuclease (Tudor-SN) is a highly conserved and ubiquitously expressed multifunctional protein, also known as SND1 (staphylococcal nuclease domain containing 1) or p100. Tudor-SN comprises one Tudor and four staphylococcal nuclease (SN) domains, with each of the SN domains lacking equivalent nuclease catalytic residues [1,2]. It was first identified in the nucleus as a co-activator of some transcriptional factors such as TFIIB, TAF40, and Stats (signal transducer and activator of transcription) [3,4,5,6,7]. Tudor-SN was further found in the cytoplasm, endoplasmic reticulum, and lipid droplets from cow and mouse mammary epithelial cells (MEC), mouse adipocytes and rat hepatocytes [8,9,10,11,12]. Tudor-SN is an intriguing protein involved in multiple and diverse cell type- and species-specific cellular processes, including transcriptional activation, RNA splicing, editing and stability, and RNAi function in maintaining normal physiological functions. Studies have shown that Tudor-SN is mainly expressed in secretory cells associating with their proliferation, apoptosis, carcinogenesis, stress response and lipid droplets biogenesis, however knowledge of its role is limited [13,14,15].

The regulation of gene expression for milk synthesis is dependent on hormonal and nutritional cues that modulate the activity of specific transcription factors in MEC [16]. The key intracellular components of the prolactin signaling pathway are the kinase Jak2 and the transcription factor Stat5 (signal transducer and activator of transcription 5), which is a central determinant of mammary gland development and function [17,18]. Stat5 is activated by tyrosine phosphorylation mainly by Jak kinases or growth factors receptor kinases, allowing their dimerization and subsequent translocation into the nucleus where it acts as a transcription factor with pleiotropic effects [19]. Stat5 is also found in one of the crucial signaling molecules downstream of other hormones, cytokine receptors, and nutrients such as amino acids and estrogen, and Stat5-mediated gene regulation is modulated by cooperation of Stat5 with cell type- and promoter-specific transcription factors as well as by interaction with transcriptional coregulators [20,21]. Tudor-SN functions as a transcriptional coactivator for Stat5-dependent gene regulation, but the mechanism through which Tudor-SN coordinates with Stat5 is not well known, furthermore, the role of Tudor-SN in the cell homeostasis of bovine MEC (BMEC) still remains unclear.

Our previous work showed that methionine (Met) is a representative amino acid that obviously activated Stat5 and mammalian target of rapamycin (mTOR) to enhance milk protein synthesis and proliferation of BMEC [22,23]. Prolactin and estrogen (E) also obviously stimulate milk synthesis and proliferation of BMEC [24,25,26]. Several reports show that Tudor-SN is a target gene of NFκB1 [27], but whether Tudor-SN is a NFκB-inducible target gene in response to amino acids and hormone stimulation is still unknown. In our previous work, we found Tudor-SN is upregulated in the nucleus of BMEC under methionine (Met) stimulation [28]. We hypothesized that Tudor-SN might play a role in the functions of BMEC in response to amino acid stimulation. Here we show that Tudor-SN is a NFκB1 target gene and coordinates with p-Stat5 to positively regulate milk protein and fat synthesis and proliferation of BMEC in responding to Met and E stimulation. This work provides further evidence that Tudor-SN is a transcriptional coactivator of Stat5 for cell homeostasis.

## 2. Results and Discussion

### 2.1. Overexpression of Tudor-SN Enhances Cell Signaling Pathways Leading to Milk Protein and Fat Synthesis and Proliferation of BMEC

**Figure 1:**
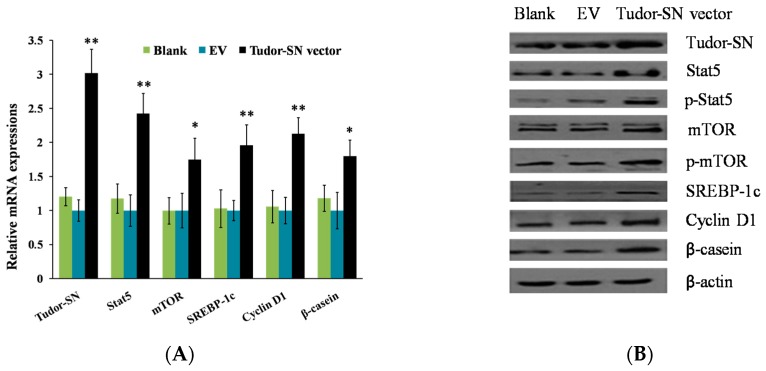

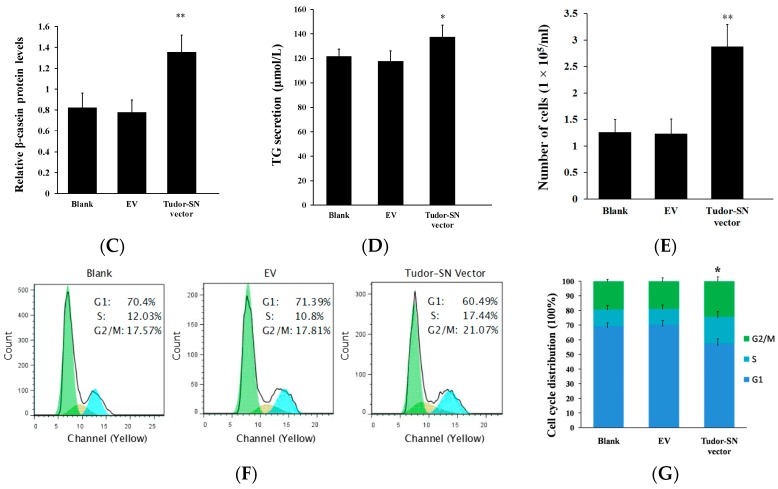
Tudor staphylococcal nuclease (Tudor-SN) overexpression upregulates cell signaling pathways leading to milk synthesis and proliferation of bovine mammary epithelial cells (BMEC). Cells were divided into three groups: Blank, untransfected cells; EV, cells transfected with an empty pGCMV-IRES-EGFP vector; and Tudor-SN vector, cells transfected with pGCMV-IRES-EGFP-Tudor-SN vector. Cells were detected 24 h after transfection. (**A**) Indicated mRNA levels were detected by qPT-PCR and normalized to β-actin; (**B**) Expression of the indicated proteins were analyzed by western blotting analysis, β-actin was used as a loading control; (**C**) The relative β-casein contents were calculated by the β-casein/β-actin relative fold change of gray scale scanning of western blotting results; (**D**) The triglyceride(TG) contents in the culture supernatants of BMEC were measured by the TG GPO-POD assay Kit (triglyceride is detected by the activities of glycerol phosphate oxidase and peroxidase); (**E**) Cell numbers were determined using a model DT CASY cell counter; (**F**,**G**) Cell cycle transition was determined by flow cytometry using a FACS Calibur. Representative histograms (**F**) and quantitation of three independent experiments (**G**) were shown, the histogram demonstrated the percentage of the cells in each phases. Blue, light blue, and green columns shows percents of cells in the G1, S, G2/M phases respectively. Data represent the mean ± standard deviation (SD). * *p* < 0.05, ** *p* < 0.01, treatment *versus* controls (EV) (Figure 1A,C–E,G), *n* = 3.

To explore the function of Tudor-SN on milk synthesis and proliferation of BMEC, we observed the effects of Tudor-SN overexpression. The pGCMV-IRES-EGFP-Tudor-SN vector was transfected into BMEC. We found that overexpression of Tudor-SN obviously increased Stat5, mTOR, SREBP-1c, Cyclin D1, and β-casein mRNA (Figure 1A) and protein (Figure 1B,C) levels in BMEC and triglyceride content (Figure 1D) in the culture supernatants of BMEC. Stat5 and mTOR phosphorylation were also promoted (Figure 1B). Cell numbers (Figure 1E) and the percentage of cells in S and G2-M phases were obviously increased whereas the percentage of cells in G1 phases were significantly decreased (Figure 1F,G). These data reveal that Tudor-SN positively regulates cell signaling pathways leading to milk protein and fat synthesis and proliferation of BMEC.

### 2.2. Gene Silencing of Tudor Staphylococcal Nuclease (Tudor-SN) Represses Cell Signaling Pathways Leading to Milk Protein and Fat Synthesis and Proliferation of Bovine Mammary Epithelial Cells (BMEC)

**Figure 2:**
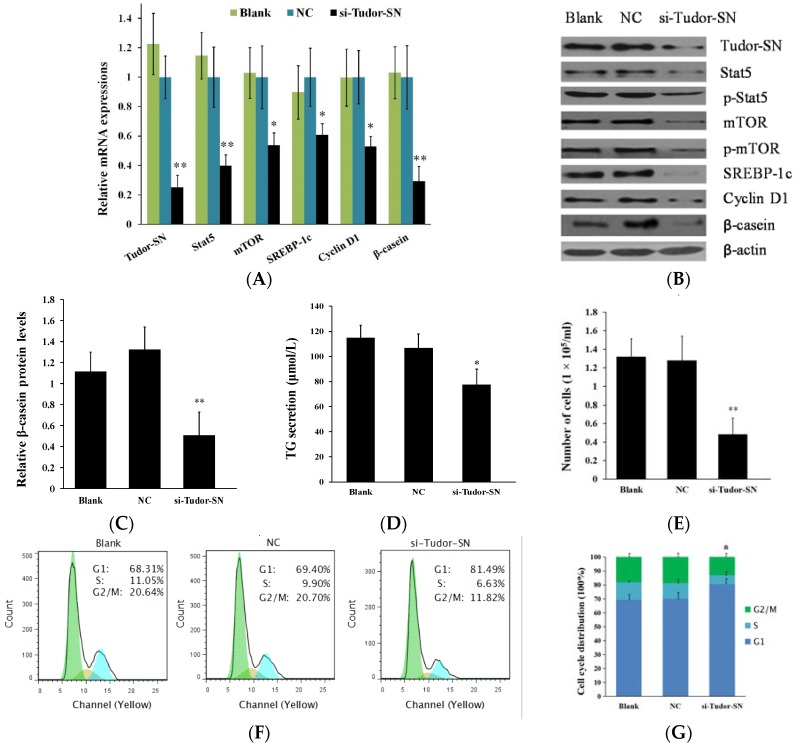
Gene silencing of Tudor-SN downregulates cell signaling pathways leading to milk synthesis and proliferation of BMEC. Cells were divided into three groups: Blank, untransfected cells; NC, cells transfected with a scrambled control RNA; and si-Tudor-SN, cells transfected with a siRNA targeting Tudor-SN. Cells were detected 24 h after transfection. (**A**) Indicated mRNA levels were detected by q RT-PCR and normalized to β-actin; (**B**) Expression of the indicated proteins were analyzed by western blotting analysis, β-actin was used as a loading control; (**C**) The relative β-casein contents were calculated by the β-casein/β-actin relative fold change of gray scale scanning of western blotting results; (**D**) The TG contents in the culture supernatants of BMEC were measured by the TG GPO-POD assay Kit; (**E**) Cell numbers were determined using a model DT CASY cell counter; (**F**,**G**) Cell cycle transition was determined by flow cytometry using a FACS Calibur. Representative histograms (**F**) and quantitation of three independent experiments (**G**) were shown, the histogram demonstrated the percentage of the cells in each phases. Blue, light blue, and green columns shows percents of cells in the G1, S, G2/M phases respectively. Data represent the mean ± standard deviation (SD). * *p* < 0.05, ** *p* < 0.01, treatment *versus* controls (NC) (Figure 1A,C,D,E), *n* = 3.

We further transfected a siRNA against Tudor-SN into BMEC. Conversely, we found that gene silencing of Tudor-SN obviously decreased Stat5, mTOR, SREBP-1c, Cyclin D1, and β-casein mRNA (Figure 2A) and protein (Figure 2B,C) levels in BMEC and triglyceride content (Figure 2D) in the culture supernatants of BMEC. Stat5 and mTOR phosphorylation (Figure 2B) were also repressed. Cell numbers (Figure 2E) and percentage of cells in S and G2-M phases were obviously decreased whereas the percentage of cells in the G1 phases was significantly increased (Figure 2F). These data provide further evidence that Tudor-SN is a positive regulator in BMEC on milk protein and fat synthesis and cell proliferation.

### 2.3. Met and Estrogen Upregulates the Expression of Tudor-SN and Interaction with p-Stat5 in the Nucleus

**Figure 3:**
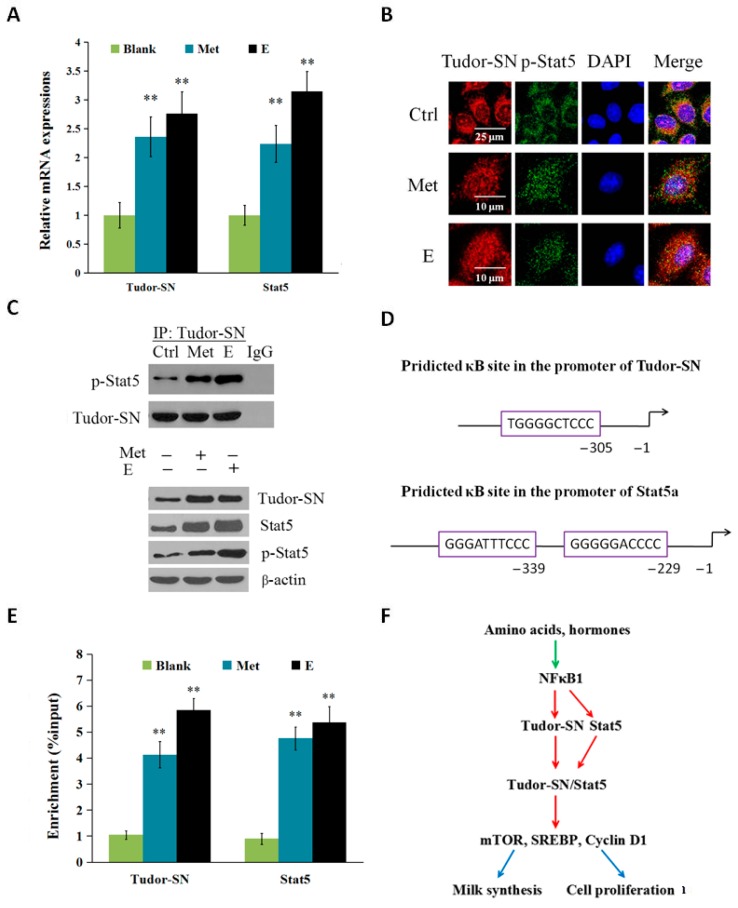
Met and estrogen increased the NFκB1-mediated gene expression of Tudor-SN and Stat5, and their localization and interaction in the nucleus. Cells were divided into three groups: Blank, normal cells; Met, cells were stimulated by Met (0.6 mmol/L); and E, cells were stimulated by E (2.72 × 10^−2^ μg/mL). Cells were detected 24 h after transfection. (**A**) Tudor-SN and Stat5 mRNA levels were detected by qPT-PCR and normalized to β-actin; (**B**) Subcellular localization of Tudor-SN and p-Stat5 24 h after Met and E stimulation were observed by immunofluorescence analysis, Tudor-SN (red), p-Stat5 (green), nuclear DNA was stained with 4′,6-diamidino-2-phenylindole (DAPI, blue). Scale bar: 25 or 10 μm; (**C**) Western blotting analysis of p-Stat5 that coimmunoprecipitated with Tudor-SN in the nuclear extracts of BMEC, Tudor-SN was used as a loading control (the **upper** panel). Protein levels of Tudor-SN, Stat5 and p-Stat5 were analyzed by western blotting analysis in cell lysates were also determined (the **lower** panel), β-actin was used as a loading control; (**D**) Predicted κB sites (GGGRNNYYCC) in the promoters of Tudor-SN and Stat5. Tudor-SN has one whereas Stat5 has two consensus κB sequences; (**E**) ChIP-qPCR analysis of the binding of p-NFκB1 to the promoters of Tudor-SN and Stat5. Data represent the mean ± standard deviation (SD). ** *p* < 0.01, treatment versus controls (blank) (Figure 3A,E, *n* = 3); (**F**) Proposed model depicting the mechanism of Tudor-SN driving milk protein and fat synthesis and cell proliferation. Under the stimulation of amino acids such as Met and hormones such as estrogen, p-NFκB1 binds to the gene promoters of Tudor-SN and Stat5 to increase their expression, and Tudor-SN binds to Stat5 in the nucleus to enhance mTOR, SREBP, and Cyclin D1 signaling pathways leading to milk synthesis and cell proliferation. Notes: The green arrow denotes positive inputs, the red arrows denote transcriptional activation, and the blue arrows denote outputs.

To determine whether Tudor-SN is regulated by environmental stimuli such as amino acids and hormones, we stimulated BMEC with Met and estrogen (E) and detected the expression of Tudor-SN and its nuclear localization and interaction with p-Stat5. Twenty-four hours after Met and E stimulation, the mRNA (Figure 3A) of Tudor-SN and Stat5 were both increased. Met and E also triggered the nuclear translocation of Tudor-SN and p-Stat5 by immunofluorescence observation (Figure 3B).

To detect whether Tudor-SN exerts its role by interacting with p-Stat5, we used Co-IP and western blotting to analyze protein levels in the nuclear extract of BMEC. Protein levels of Tudor-SN, Stat5 and p-Stat5 were all upregulated in cell lysates, and the interaction of Tudor-SN with p-Stat5 in the nucleus was enhanced by Met and E stimulation (Figure 3C).

To further investigate whether the gene expression of Tudor-SN is induced by NFκB, we exploited ChIP-qPCR analysis. We detected the binding of p-NFκB1 to the gene promoters of *Tudor-SN* and *Stat5* in cells treated with Met (0.6 mmol/L) or E (2.72 × 10^−2^ μg/mL). We first predicted the consensus κB binding site (GGGRNNYYCC, R: purine, Y: pyrimidine, N: any base) in the promoters of *Tudor-SN* and *Stat5a* (Figure 3D), and verified by qRT-PCR the immunoprecipates in the ChIP assays that employed antibodies against p-NFκB1. We then measured the changes in the enrichment of the binding sequences in ChIP assays using the antibody against p-NFκB1 for BMECs treated with Met and E. The enrichment was dramatically higher in cells treated with Met and E compared with the control (Figure 3E). These data suggest that both Tudor-SN and Stat5 are NFκB1 target genes in response to environmental stimuli such as amino acids and hormones.

### 2.4. Discussion

Lots of experiments in our laboratory confirm that primary MEC (from mouse, goat and cow) express β-casein in the culture of DF12 and 10% FBS with no addition of prolactin or glucocorticoids. Ordinarily, we use BMEC in 0 to 6 passages in the culture of DF12 and 10% FBS for cell proliferation to gain enough cells. BMEC in 7 to 15 passages in the same culture differentiates thoroughly with no additional differentiation treatment, and are used for experimental tests. BMEC over 15 passages are discarded for they begin to lose proliferation ability and sensitivity to hormones, amino acids, and transfection of plasmids.

Our gene function studies suggest that Tudor-SN positively regulates Stat5, mTOR, SREBP-1, and Cyclin D1 signaling pathways. Tudor-SN has been reported as coactivators of Stat5 [6,9]. Jak-Stat and mTOR pathways have been confirmed to control milk synthesis and proliferation of BMEC and Stat5a positively regulates mTOR pathway in BMEC [22,29]. mTORC1 promotes the function of SREBP, a master regulator of lipo- and sterolgenic gene transcription [30,31] and SREBP is a known key regulator on milk fat synthesis [32,33]. mTORC1 also regulates Cyclin D1 to control cell proliferation [34,35]. Recently, many reports indicate that Tudor-SN is a key regulator of cell proliferation [36,37,38]. These reports, together with our experimental results, suggest that Tudor-SN is a positive regulator of milk protein and fat synthesis and proliferation of BMEC by affecting Stat5 and mTOR pathways. To our best knowledge, this is the first report that Tudor-SN regulates mTOR pathways for cell homeostasis. The fact that Tudor-SN binds to several hundred gene promoters gives the clue that it might regulate mTOR gene transcription [27]; further research is needed to reveal the profile of Tudor-SN target genes.

By immunofluorescence observation, we found both Tudor-SN and p-Stat5a are triggered by Met and E for nuclear translocation. Further using the Co-IP technique, we demonstrated that Tudor-SN binds to p-Stat5a in the nucleus, in agreement with previous results [6,9], and provides further evidence that Tudor-SN is a coactivator of Stat5 for gene transcription. We show that this interaction is enhanced through amino acids (such as Met) and hormones (such as E), suggesting that the interaction between Tudor-SN and p-Stat5a is affected by environmental stimuli and is important for the cell signaling network. How Tudor-SN affects the activity of Stat5 is not fully understood. A report indicates that Tudor-SN is highly phosphorylated during the cell cycle [37,38] and is a potential substrate of Cdk2/4/6, but we still do not know the subcellular localization of phosphorylated Tudor-SN and function of Tudor-SN phosphorylation on the coactivation of Stat5, and which is the upstream molecule to activate this phosphorylation. Recently, reports found that Tudor-SN binds to many signaling molecules such as metadherin, Cdk4/6, and many core components of stress granules [39,40,41], suggesting that it is a multifunctional protein, related to multiple and diverse cell type- and species-specific cellular processes. Further interactome study on Tudor-SN and its phosphorylated form in different cell departments is needed to understand the mechanism of its pleiotropic effects.

We observed that Met or E stimulation triggers the Tudor-SN or Stat5 modification (phosphorylation), but we still do not know whether the increase of Stat5 or mTOR phosphorylation is because of the increase of protein level or the modification. We show that Met or E stimulate the transcription of Tudor-SN and Stat5, furthermore the phosphorylation of Tudor-SN and Stat5 are also enhanced. We speculate that these enhanced phosphorylations are due to both upregulated gene expression and direct phosphorylation by other kinases via an unknown mechanism.

In our study, we also showed that Tudor-SN and Stat5 are both activated by Met and E via NFκB1 target gene expression. Previous report revealed that NFκB binds to the Tudor-SN promoter, in agreement with our results. TNFα, an activator of NFκB, induced Tudor-SN expression [14]. NFκB is a ubiquitous transcription factor involved in cell proliferation and differentiation [42,43]. Our previous study found that Met and E triggered NFκB1 phosphorylation and enhanced the binding of p-NFκB1 to the Stat5 gene promoter (data not shown), and in this study we also validated the interaction of p-NFκB1 and Tudor-SN gene promoter. We still do not know the mechanism by which NFκB senses Met and E. The fact that NFκB1 mediates the gene expression of Tudor-SN and Stat5 also suggests that NFκB1 as an upstream molecule of Tudor-SN and Stat5 is a positive regulator on the milk synthesis and proliferation of BMECs.

## 3. Materials and Methods

### 3.1. Cell Preparation and Treatments

Primary BMEC was purified and cultured as previously reported [28]. Briefly, blocks (~1 mm^3^) of fresh mammary gland tissues from mid-lactation dairy cows were moved into the bottom of the cell culture bottles, and incubated at 37 °C, 5% CO_2_ for 3 h, then DMEM/F-12 medium supplemented with 10% FBS, 100 U/mL penicillin and 100 μg/mL streptomycin was gently added into the culture bottles just covering tissue blocks. The medium was replaced every 3 days. About 15–30 days later, fibroblast and BMEC mixed cells covered the bottom of the cell culture bottle. BMECs were further purified with 0.25% trypsin and 0.02% EDTA solution. The cell purity and milk protein synthesis ability of these BMEC were evaluated by the immunofluorescence observation of cytokeratin 18 and β-casein, and the purified BMECs in 7–15 passages were used for further experiments. To investigate the influence of methionine (Met) and estrogen (E) (estradiol, E2) on Tudor-SN and Stat5 expression in BMEC, cells in DMEM/F-12 medium (containing 0.12 mmol/L Met) were treated with Met (0.6 mmol/L) or E (2.72 × 10^−2^ μg/mL) for 24 h, and processed for further analysis.

### 3.2. Cell Number Assay

Cell viability and numbers were determined as in a previous report [28] using a CASY TT Analyser System (Scharfe System GmbH, Reutlingen, Germany). This system was used for automated cell counting and for measurement of viability according to the size of cells. Briefly, cells were digested with trypsin and diluted (1:100) with CASY electrolyte solution before examination, then 100 μL aliquots were aspirated into a capillary and passed through a precision measuring pore. Vital cells with intact cell membranes were considered as an electrical isolator and the resistance measurement reflects the true size of the living cell, while dead cells were identified by the size of their nucleus. The dimension of cell debris was less than 7.63 mm, the dimension of dead cells was between 7.63 and 11.75 mm, and the dimension of vital cells was larger than 11.75 mm.

### 3.3. Analysis of Cell Cycle Progression

The proportion of BMEC in various different cell cycle phases was measured by flow cytometric analysis [44,45]. Cells were plated at 3 × 10^4^ cells/cm^2^ and incubated in six-well culture plates with experiment culture medium without FBS for at least 36 h to synchronize the cell cycle. Twenty-four hours after treatment, cells were washed with cold PBS, trypsinized, and collected by centrifugation, and then fixed with 70% cold ethanol at 4 °C overnight, then re-suspended twice with PBS containing 0.1 mg/mL RNase A and 5 μg/mL propidium iodide (PI). After incubation for 30 min at room temperature in the dark, the cells were washed in 500 mL of PBS and introduced into a FACS Calibur (Guava easyCyte 5HT flow-cytometer System, Millipore, Bedford, MA, USA) for cell cycle analyses. The raw data were analyzed by FlowJo software (Tree Star Inc, Ashland, OR, USA).

### 3.4. Detection of Triglyceride Secretion

Cell-free supernatants were subsequently employed for triglyceride assay by using the Triglyceride (TG) GPO-POD assay Kit (Applygen Tech Inc., Beijing, China) according to the manufacturer’s instructions and a previous report [44].

### 3.5. RNA Extraction and Real Time PCR

TRIzol reagent was used to extract total RNA of BMEC. For cDNA synthesis, total RNA was used to generate cDNA using Reverse Transcription System (TaKaRa, Dalian, China) according to the manufacturer’s protocols. RT-PCR were performed using SYBR PrimeScript RT-PCR Kit (TaKaRa) by the ABI PRISM 7300 Real-Time PCR System (Applied Biosystems, Foster City, CA, USA). β-actin was used as a normalization control. The oligonucleotide sequences of primers for the target genes were as follows: Tudor-SN, sense 5′-GAGCAAGCGAAAGCATCT-3′, antisense 5′-ACCGTGACCAGGTAGTAATCT-3′; Stat5, sense 5′-GTCCCTTCCCGTGGTTGT-3′, antisense 5′-CGGCCTTGAATTTCATGTTG-3′; mTOR, sense 5′-ATGCTGTCCCTGGTCCTTATG-3′, antisense 5′-GGGTCAGAGAGTGGCCTTCAA-3′; SREBP1, sense 5′-AGTAGCAGCGGTGGAAGT-3′, antisense 5′-GCAGCGGCTCTGGATT-3′; cyclinD1, sense 5′-CCGTCCATGCGGAAGATC-3′, antisense 5′-CAGGAAGCGGTCCAGGTAG-3′; β-casein, sense 5′-AACAGCCTCCCACAAAAC-3′, antisense 5′-AGCCATAGCCTCCTTCAC-3′; β-actin, sense 5′-AAGGACCTCTACGCCAACACG-3′, antisense 5′-TTTGCGGTGGACGATGGAG-3′. Data analysis was performed by the 2^−ΔΔ*C*^_T_ method.

### 3.6. Tudor-SN Overexpression

The full-length of Tudor-SN coding region was obtained by PCR using the specific primer designed with particular restriction enzyme sites and protective bases. Primers sequences were as follows, sense: 5′-GAAGATCTATGGCCTCCTCCGCGCAGAGC-3′ (Bgl II) and antisense: 5′-GCGTCGACTGGAAGGAGAGGGCAGGTC-3′ (Sal I) (NCBI accession number: NM_205784.1). The full length of Tudor-SN fragment was then subcloned into pGCMV-IRES-EGFP vector (GenePharma, Shanghai, China), and subjected to sequencing for verification.

BMEC transfections of recombinant and empty vector plasmids were performed using Lipofectamine 2000 (Invitrogen, Carlsbad, CA, USA). Non-transfected BMEC was served as additional controls. Briefly, 1 μg plasmid DNA and 2.5 μL Lipofectamine 2000 were diluted in 200 μL OPTI-MEMI medium (Life Technologies, Grand Island, NY, USA) and incubated at room temperature for 20 min to form the DNA-Lipofectamine 2000 mixtures, then incubated with BMEC in serum- and antibiotic-free medium at 37 °C for about 4–6 h. After transfection, the medium was replaced with experimental culture medium and cultured for 24 h.

### 3.7. siRNA and Transfection

The Tudor-SN siRNA and negative control RNA-oligonucleotides were purchased from Shang Hai GenePharma. The siRNA sequences targeting Tudor-SN were as follows: sense 5′-GCGAUCCUCUCACUAUGAUTT-3′, antisense 5′-AUCAUAGUGAGAGGAUCGCTT-3′; the nonrelevant scrambled control sequence were as follows: sense 5′-UUCUCCGAACGUGUCACGUTT-3′, antisense 5′-ACGUGACACGUUCGGAGAATT-3′. BMEC transfections of Tudor-SN siRNA, and negative control siRNAs were performed using Lipofectamine 2000. Non-transfected BMEC was served as an additional control. Briefly, BMEC were trypsinized and plated in 6-well plates at 1 × 10^6^ cells per well in DMEM/F-12 medium. For each well, 1 μg siRNA and 2 μL Lipofectamine 2000 were diluted in 200 μL OPTI-MEMI medium and incubated at room temperature for 20 min, then incubated with BMEC in serum- and antibiotic-free medium at 37 °C about 4–6 h. After transfection, the medium was replaced with experimental culture medium and cultured for 24 h.

### 3.8. Western Blotting Analysis

Western blot analysis was performed as described previously [23,29]. Briefly, 30 μg of protein equivalents of whole-cell extracts was separated on 10% SDS-PAGE. Gels were transferred to nitrocellulose membranes, then the membranes were blocked in 5% skim milk in pH 7.5 Tris-buffered saline with 0.1% Tween (TBST) for 2 h at 37 °C, then incubated with primary antibodies in 5% skim milk/TBST overnight at 4 °C, and then incubated with horseradish peroxidase-conjugated IgG in 5% skim milk/TBST for 1.5 h at 37 °C. The chemiluminescence detection of HRP-conjugated secondary antibodies was performed using Super ECL plus (ApplyGEN, Beijing, China).

Primary antibodies against the following proteins were used: Tudor-SN (ab 71186), p-NFκB1 p105/p50 (S337) (ab28849), Abcam, Cambridge, MA, USA; mTOR (#2983), p-mTOR (Ser2448) (#5536), and p-Stat5 (Tyr694) (#9359), Cell Signaling Technology, Beverly, MA, USA; cyclinD1 (sc-753), Stat5 (sc-28685), SREBP-1c (the mature form, 68 kD) (sc-365513), β-actin (sc-47778), Santa Cruz, CA, USA; β-casein (Abbiotec, San Diego, CA, USA, 251309).

### 3.9. Immunofluorescence

Immunofluorescence assay was carried out as described previously [29]. BMEC was probed with primary antibodies against the following: β-casein (1:50, Abbiotec, 251309), cytokeratin 18 (Santa Cruz, CA, USA, sc-51582), Tudor-SN (Abcam, ab 71186), p-Stat5 (Tyr694) (Cell Signaling Technology, #9359). Alexa Fluor 488 or Alexa Fluor 647 secondary antibodies (ZSGB-BIO, Beijing, China) were used to detect primary antibodies. Following PI or DAPI staining, the cells were imaged under a TCS-SP2 AOBS confocal microscope (Leica, Heidelberg, Germany).

### 3.10. Co-IP Assay

The nuclear fractions of BMEC were separated and extracted by using NE-PER Nuclear and Cytoplasmic Extraction Kit (P0028), Beyotime Institute of Biotechnology, Shanghai, China. Proteins were immunoprecipitated using a Pierce Co-Immunoprecipitation (Co-IP) Kit (Pierce, IL, USA) according to the manufacturer’s protocol. Briefly, 200 μg of nuclear protein lysates were applied to columns containing 20 μg immobilized Tudor-SN antibody covalently linked to an amine-active resin and incubated overnight at 4 °C. As negative controls, control (preimmune) IgG was used to immunoprecipitate the sample lysate. The IP complexes along with the negative controls were subjected to western blotting analysis.

### 3.11. ChIP-PCR and ChIP-qPCR

An anti-p-NFκB1 antibody-based chromatin immunoprecipation (ChIP) was performed using an EpiQuikTM Chromatin Immunoprecipitation Kit (#P-2002, Epigentek, Farmingdale, NY, USA) with antibodies against p-NFκB1 (S337) (Abcam, ab28849). An antibody against RNA polymerase was used as the positive control and IgG as the negative control. The predicted κB sites in Tudor-SN gene promoter is TGGGGCTCCC, and the qPCR primers for Tudor-SN are: sense 5′-TGGCGTTCCCAGCTCGCTCTA-3′, antisense 5′-GGAGGTGTAGTGGGAAGGAAAGGAC-3′. The predicted κB sites in Stat5a gene promoter are GGGATTTCCC and GGGGGACCCC, and the qPCR primers for Stat5a are: sense 5′-CTCCAACCCTTTCCTCCTCCCTTTT-3′, antisense 5′-AGCACGGCTGCGAGGCGG-3′. The oligonucleotide sequences of primers to amplify Tudor-SN and Stat5 gene promoters were designed and sequenced, then the amplification of predicted sequence using these primers were verified by ChIP-PCR. For ChIP-qPCR, the data represent the percentage of input that is immunoprecipitated.

### 3.12. Statistical Analysis

Data are presented as means standard deviation from at least three separate experiments. Statistics and differences between groups were analyzed using Student’s *t*-tests (SPSS 17.0 software, SPSS, Chicago, IL, USA). Differences with *p* < 0.05 were considered statistically significant, and *p* < 0.01 highly significant.

## 4. Conclusions

Together, these results reveal that Tudor-SN is a positive regulator of milk protein and fat synthesis and proliferation of BMEC. Tudor-SN positively regulates Stat5, mTOR, SREBP-1, and Cyclin D1. Amino acids and hormones such as Met and E trigger NFκB target Tudor-SN gene expression and nuclear localization, furthermore they enhance the interaction of Tudor-SN with p-Stat5 in the nucleus (Figure 3F).
